# Identification and Bioactivity Analysis of a Novel *Bacillus* Species, *B. maqinnsis* sp. nov. Bos-x6-28, Isolated from Feces of the Yak (*Bos grunniens*)

**DOI:** 10.3390/antibiotics13121238

**Published:** 2024-12-23

**Authors:** Qiang Ma, Xin Xiang, Yan Ma, Guangzhi Li, Xingyu Liu, Boai Jia, Wenlin Yang, Hengxia Yin, Benyin Zhang

**Affiliations:** 1 College of Eco-Environmental Engineering, Qinghai University, Xining 810016, China; ys220901j10478@qhu.edu.cn (Q.M.); 2023970035@qhu.edu.cn (X.X.); ys240860020139@qhu.edu.cn (G.L.); ys240860010125@qhu.edu.cn (X.L.); 230710044121@qhu.edu.cn (B.J.); 230710024131@qhu.edu.cn (W.Y.); 2 State Key Laboratory of Plateau Ecology and Agriculture, Qinghai University, Xining 810016, China; 3 College of Eco-Environmental Engineering, Qinghai Vocational and Technical University, Xining 810016, China; 210710020325@qhu.edu.cn

**Keywords:** yak, intestinal bacteria, *Bacillus*, polyphasic taxonomy, biosynthetic gene clusters, bioactivity

## Abstract

**Background:** The identification of novel bacterial species from the intestines of yaks residing on the Qinghai–Tibet Plateau is pivotal in advancing our understanding of host–microbiome interactions and represents a promising avenue for microbial drug discovery. **Methods:** In this study, we conducted a polyphasic taxonomic analysis and bioactive assays on a *Bacillus* strain, designated Bos-x6-28, isolated from yak feces. **Results:** The findings revealed that strain Bos-x6-28 shares a high 16S rRNA gene sequence similarity (98.91%) with *B. xiamenensis* HYC-10^T^ and *B. zhangzhouensis* DW5-4^T^, suggesting close phylogenetic affinity. Physiological and biochemical characterizations demonstrated that Bos-x6-28 could utilize nine carbon sources, including D-galactose, inositol, and fructose, alongside nine nitrogen sources, such as threonine, alanine, and proline. Analysis of biochemical markers indicated that Bos-x6-28’s cell wall hydrolysates contained mannose, glucose, and meso-2,6-diaminopimelic acid, while menaquinone-7 (MK-7), phosphatidylethanolamine (PE), phosphatidylcholine (PC), and phosphatidylglycerol (DPG) were found in the cell membrane. The primary cellular fatty acids included C16:0 (28.00%), cyclo-C17:0 (19.97%), C14:0 (8.75%), cyclo-C19:0 (8.52%), iso-C15:0 (5.49%), anteiso-C15:0 (4.61%), and C12:0 (3.15%). Whole-genome sequencing identified a genome size of 3.33 Mbp with 3353 coding genes. Digital DNA–DNA hybridization (dDDH) and average nucleotide identity (ANI) analyses confirmed Bos-x6-28 as a novel species, hereby named *B. maqinnsis* Bos-x6-28 (MCCC 1K09379). Further genomic analysis unveiled biosynthetic gene clusters encoding bioactive natural compounds, including β-lactones, sactipeptides, fengycin, and lichenysin analogs. Additionally, in vitro assays demonstrated that this strain exhibits antibacterial and cytotoxic activities. **Conclusions:** These findings collectively indicate the novel *Bacillus* species *B. maqinnsis* Bos-x6-28 as a promising source for novel antibiotic and antitumor agents.

## 1. Introduction

The gut microbiota plays a pivotal role in host health and environmental adaptation, facilitating not only digestive and metabolic processes but also modulating the immune system through diverse mechanisms to bolster pathogen defense [1,2]. Recent advancements in high-throughput sequencing and metagenomics have significantly enhanced our understanding of the gut microbiome’s composition and functional capacities [3,4]. These developments have shed light on the dynamic shifts in microbial communities in response to environmental fluctuations and their involvement in disease processes. Although high-throughput and metagenomic approaches have unveiled unparalleled microbial diversity and functional potential without the need for culturing, molecular methods alone have inherent limitations. To pinpoint specific bacterial taxa critical for maintaining health or linked to disease, it is imperative to incorporate culture-dependent techniques.

Culture-independent metagenomic studies have underscored a strong association between gut microbiota and host health and disease [5,6]. Nevertheless, it has long been assumed that most gut microbes cannot be cultured under laboratory conditions, leaving their phenotypic and functional profiles largely unknown [7,8]. However, increasing findings challenge this notion, demonstrating that numerous gut microbes are indeed culturable and often reveal greater species diversity than culture-independent methods suggest [9]. For example, Ito et al. isolated eight novel bacterial species from the human gut microbiota within the Actinobacteria and Firmicutes phyla using traditional culture techniques [10]. By integrating culturing with next-generation sequencing, they established that culturable operational taxonomic units (OTUs) comprise nearly 50% of the fecal genome [11]. Lagier et al. pioneered a “culturomics” approach, employing 212 culture conditions, MALDI-TOF mass spectrometry, and 16S rRNA gene sequencing to identify 1057 gut microbial species, 197 of which are potentially new species [12]. Moreover, Rettedal et al. observed that 88% of microbial taxa at the family level could be cultured, with culture-dependent methods detecting 40% more OTUs than culture-independent approaches [13], highlighting the advantages of cultivation in studying gut microbiota diversity.

Metagenomic sequencing of the gut microbiota reveals its significant potential for synthesizing a vast array of biosynthetic natural products. This microbial “dark matter” represents a critical reservoir for novel drug discovery [14,15,16]. For instance, Ma et al. recently isolated and identified four antimicrobial compounds, including *Gordonia* sp., a resident of the intestinal tract of *Periplaneta americana* [17]. Additionally, two structurally unique compounds, Coprisamides A and B, exhibiting notable induction activity on quinone reductase, were isolated from the gut microbiota of the dung beetle *Copris tripartitus* [18]. These findings suggest that active natural products derived from animal gut microbiota hold substantial untapped potential for research. Further exploration of these bioactive molecules may facilitate the discovery of novel therapeutic agents.

The yak (*Bos grunniens*), an essential livestock species on the Tibetan Plateau, has adapted to extreme alpine conditions through unique physiological and metabolic traits [19]. Growing evidence suggests that the yak gut microbiota is integral to both adaptation to these harsh conditions and health maintenance [20,21,22]. Our previous study indicated that gut microbiota enhances yak adaptability to high-altitude environments by modulating metabolic functions, revealing a wealth of uncharacterized microbial resources [23]. In addition to influencing yak health through their metabolic functions, gut microbiota in yaks also maintain health via immune and neural-activation pathways. For instance, short-chain fatty acids (SCFAs) produced by *Ruminococcus* can enhance host immunity and inhibit pathogen colonization by regulating gut pH [24]. Notably, this bacterial genus was also identified in our previous studies on yak gut bacterial diversity [23]. Yaks have evolved a microbial strategy, supported by certain probiotic bacteria, to sustain their health in the harsh plateau environment. Currently, multiple *Bacillus* species with antibacterial activity have been isolated from yak gut contents or feces, including *B. licheniformis*, *B. pumilus*, *B. subtilis*, *B. veleis*, and *B. cereus* [25,26,27,28]. Many of these Bacillus strains exhibit probiotic properties. For example, *B. pumilus* DX24, *B. licheniformis* ZLBB-1995, and *B. subtilis* 18, isolated from yak feces, have demonstrated growth-promoting effects in mice [25,29,30]. Additionally, *B. licheniformis* ZLBB-1995 has been shown to influence the structure and metabolism of the host gut microbiota [29]. These findings suggest that Bacillus species from the yak gut are not only valuable sources of antimicrobial agents but also hold significant potential as probiotics. From the feces of free-ranging yaks in high-altitude regions, we isolated a *Bacillus* strain, Bos-x6-28, preliminarily identified as a novel species within the *Bacillus* genus. In this study, we employed polyphasic taxonomy to characterize this strain at physiological and molecular levels and used the antiSMASH online tool to analyze its secondary metabolite biosynthetic gene clusters. Finally, we evaluated its antibacterial and antitumor activities. This research is of considerable importance in advancing our understanding of yak gut microbiota diversity and functional potential.

## 2. Results

### 2.1. Identification of Strain Bos-x6-28 Based on 16S rRNA Gene Sequence Analysis

**Figure 1:**
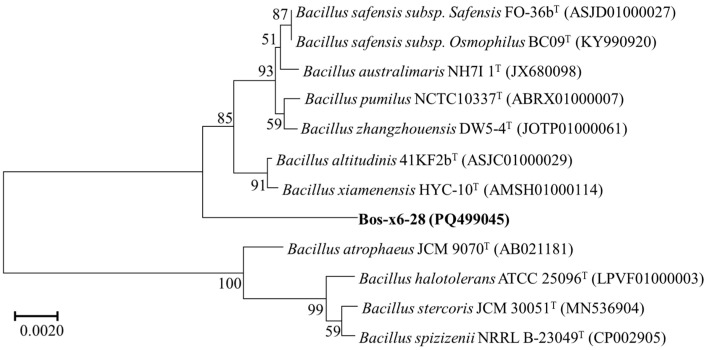
Phylogenetic analysis of strain Bos-x6-28 based on 16S rRNA gene sequence using the neighbor-joining (NJ) method. The sequence numbers in parentheses represent the GenBank accession numbers of the corresponding strains. The scale bar indicates a 0.02 nucleotide divergence per site.

The 16S rRNA gene of strain Bos-x6-28 was amplified and sequenced, resulting in a 1549 bp sequence. Comparative analysis in the EzBioCloud database indicated that Bos-x6-28 shares the highest similarity, at 98.91%, with *Bacillus xiamenensis* HYC-10^T^ and *Bacillus zhangzhouensis* DW5-4^T^. Furthermore, a phylogenetic tree was constructed using the 16S rRNA gene sequences of Bos-x6-28 and its closest related strains (Figure 1). The phylogenetic analysis revealed that, although Bos-x6-28 is most closely related to *B. xiamenensis* HYC-10^T^ in terms of genetic distance, it forms a separate clade, indicating that Bos-x6-28 may represent a novel species within the *Bacillus* genus.

### 2.2. Morphological Analysis of Strain Bos-x6-28

**Figure 2:**
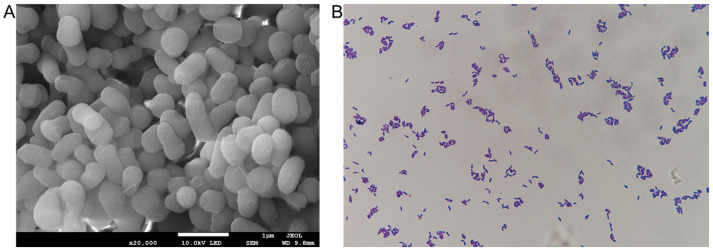
Microscopic structure of strain Bos-x6-28. (**A**) Scanning electron microscopy (SEM) at 20,000× magnification, showing the detailed ultrastructure of Bos-x6-28. (**B**) Light microscopy at 1000× magnification after Gram staining, illustrating cellular morphology and Gram characteristics.

After being cultured on ISP2 medium for 3 days, colonies of strain Bos-x6-28 appeared brown, with a dry, wrinkled surface and smooth edges. Examination of the ultrastructure through electron microscopy (Figure 2) revealed that the spores of Bos-x6-28 are rod-shaped. Observation under a light microscope following Gram staining showed that Bos-x6-28 stained purple, identifying it as a Gram-positive bacterium.

### 2.3. Physiological and Biochemical Characteristics of Strain Bos-x6-28

Tolerance experiments revealed that strain Bos-x6-28 can grow across a temperature range of 10–40 °C, with an optimal growth temperature of 35 °C (Table 1, Supplementary Figure S1). Additionally, it can tolerate NaCl concentrations between 0% and 5%. The strain grows within a pH range of 4–10, with an optimal growth pH of 7.0. These characteristics are highly consistent with the similar type strains *B. xiamenensis* HYC-10^T^ and *B. zhangzhouensis* DW5-4^T^ (Table 1).

Carbon source utilization tests showed that Bos-x6-28 can utilize a variety of sugars, including D-galactose, inositol, fructose, uranose, D-mannose, glucose, xylose, and ribose. In comparison, the type strains HYC-10^T^ and DW5-4^T^ utilize a slightly broader range of sugars. For instance, among the 12 tested sugars, HYC-10^T^ could utilize all except D-mannose and inositol, while DW5-4^T^ could not utilize rhamnose, inositol, and sorbitol (Table 1). In terms of nitrogen source utilization, Bos-x6-28, similarly to the two type strains, can utilize nine amino acids, including threonine, alanine, proline, asparagine, serine, arginine, tyrosine, glutamate, and glycine (Table 1). Further analysis revealed that Bos-x6-28 possesses lipase activity but lacks the capability to produce H_2_S and hydrolyze starch, aligning with the characteristics of the type strains.

Enzyme activity tests demonstrated that Bos-x6-28 exhibits various enzyme activities, including alkaline phosphatase, lipase (C4), esterase lipase (C8), leucine arylamidase, valine arylamidase, trypsin, cystine arylamidase, chymotrypsin, acid phosphatase, C14 lipase, β-galactosidase, α-mannosidase, α-fucosidase, and α-glucosidase. The enzyme activity profile of Bos-x6-28 showed the highest similarity to that of the strain type DW5-4^T^ (Table 1).

### 2.4. Chemical Taxonomic Characteristics of Strain Bos-x6-28

**Figure 3:**
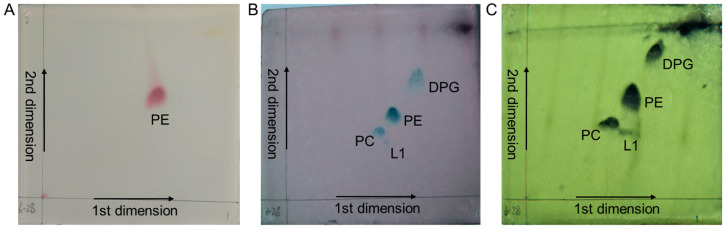
Two-dimensional TLC analysis of phospholipid components in strain Bos-x6-28. (**A**) Ninhydrin staining; (**B**) Anisaldehyde staining; (**C**) Phosphomolybdic acid staining.

Thin-layer chromatography (TLC) analysis identified mannose and glucose as the primary sugars in the whole-cell hydrolysates of strain Bos-x6-28, with meso-2,6-diaminopimelic acid as the characteristic amino acid component. Menaquinone analysis of the cell membrane via liquid chromatography-tandem mass spectrometry (LC-MS/MS) revealed a molecular ion peak [M + H]^+^ at 649.2742 (Supplementary Figure S2), which, based on molecular weight and retention time, was identified as menaquinone-7 (MK-7). In addition, the phospholipid composition of the cell membrane was examined using two-dimensional TLC and subsequently stained with ninhydrin, molybdophosphoric acid, and anisaldehyde (Figure 3). The analysis showed that the primary phospholipids included phosphatidylethanolamine (PE), phosphatidylcholine (PC), diphosphatidylglycerol (DPG), and an unidentified phospholipid (L1).

Gas chromatography (GC) of the fatty acid profile in Bos-x6-28 cells revealed that the cell membrane contains significant proportions of various fatty acids, including C16:0 (28.00%), cyclo-C17:0 (19.97%), C14:0 (8.75%), cyclo-C19:0 (8.52%), iso-C15:0 (5.49%), anteiso-C15:0 (4.61%), and C12:0 (3.15%) (Table 2). The fatty acid composition and relative abundances in Bos-x6-28 exhibited considerable differences compared to those observed in the two strain types [31,32].

### 2.5. Genome Sequencing and Comparative Analysis of Strain Bos-x6-28

**Figure 4:**
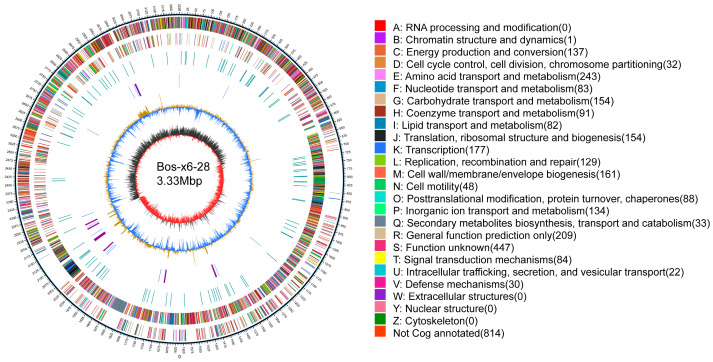
Circular genome map of strain Bos-x6-28. The outermost ring denotes the genomic size, with each tick mark representing 5 kb. The second and third rings display genes on the positive and negative strands, respectively, with different colors indicating various COG functional classifications. The fourth ring represents repeat sequences. The fifth ring shows tRNA (blue) and rRNA (purple) genes. The sixth ring illustrates GC content, where light yellow areas indicate regions with GC content higher than the genome’s average, with peak heights corresponding to the extent of deviation from the mean; blue areas denote regions with GC content below the genomic average. The innermost ring represents the GC-skew, with dark gray indicating regions where G content exceeds C content, and red indicating regions where C content exceeds G content.

Whole-genome sequencing of strain Bos-x6-28 was conducted on the PacBio platform, producing a total of 1,130,375 clean reads after filtration. The genome assembly yielded a total size of 3.33 Mbp with a GC content of 40.22%, organized into three scaffolds. Scaffold 1 represents the chromosomal DNA, measuring 3,225,051 bp, while scaffolds 2 and 3 correspond to plasmid genomes of 58,834 bp and 45,686 bp, respectively (Table 3). Gene prediction using Prodigal v2.6.3 identified 3353 coding sequences, totaling 2,841,843 bp, which constitutes 85.35% of the genome (Figure 4).

To determine the precise taxonomic positioning of Bos-x6-28, digital DNA–DNA hybridization (dDDH) and average nucleotide identity (ANI) analyses were calculated, comparing Bos-x6-28 to strain types HYC-10^T^ and DW5-4^T^. The analysis revealed that Bos-x6-28 shares the highest dDDH and ANI values with the strain type DW5-4^T^, at 54.7% and 85.88%, respectively (Table 4). Both values fall below the dDDH and ANI thresholds of 70% and 95%, respectively, which are conventionally applied to delineate new bacterial species [33,34]. These results robustly support the conclusion that Bos-x6-28 constitutes a novel species within the genus *Bacillus*.

**Figure 5:**
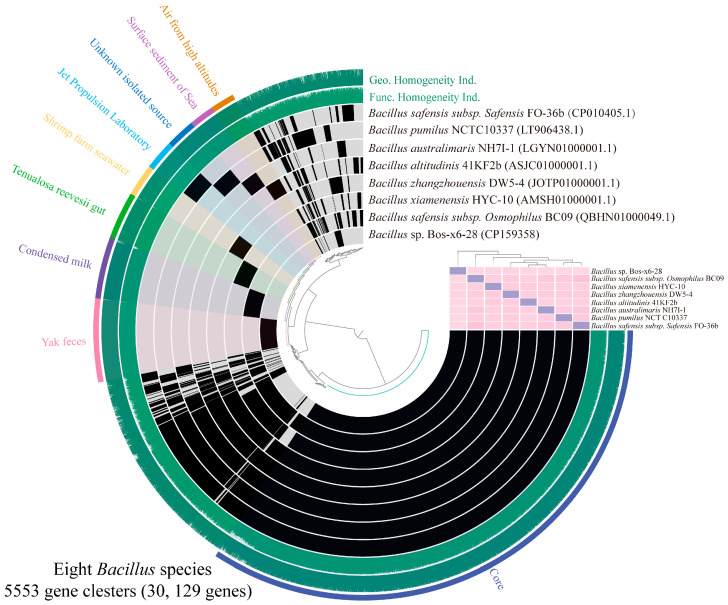
Comparative genomic analysis of strain Bos-x6-28 and its closely related species. The outermost ring represents the strain origin and shared core gene clusters, followed by the geometric homogeneity index and functional homogeneity index. Subsequent rings display data for *B. safensis* subsp. *safensis* FO-36b, *B. pumilus* NCTC10337, *B. australimaris* NH7I-1, *B. altitudinis* 41KF2b, *B. zhangzhouensis* DW5-4, *B. xiamenensis* HYC-10, *B. safensis* subsp. *osmophilus* BC09, and Bos-x6-28. The heat map shows the ANI values among these strains, with all ANI values below 95% (indicated in pink), highlighting the genomic divergence among the strains.

Through comparative genomic analysis with closely related reference strains, we identified a total of 30,129 genes across the genomes of strain Bos-x6-28 and seven Bacillus strains from diverse sources, which were grouped into 5553 orthologous gene clusters (Supplementary Table S1). Among these, 21,181 core genes shared across all strains were organized into 2553 gene clusters (Figure 5). Moreover, ANI analysis demonstrated that the similarity between Bos-x6-28 and other strains remained consistently below 95%, reinforcing its classification as a novel *Bacillus* species.

### 2.6. Analysis of Secondary Metabolite Biosynthetic Potential of Strain Bos-x6-28

The secondary metabolite biosynthetic potential of *Bacillus* strain Bos-x6-28 was examined using the antiSMASH online tool, which identified eight distinct biosynthetic gene clusters spanning six classes within its genome (Table 5). These clusters include two associated with β-lactone-type compounds, one for terpene biosynthesis, one for Type III polyketides (T3PKS), a siderophore cluster, and two clusters linked to ribosomally synthesized and post-translationally modified peptides (RiPPs), specifically an RRE-containing peptide and a sactipeptide, as well as one cluster encoding for non-ribosomal peptide synthetase (NRPS).

Comparative analyses with known gene clusters revealed that clusters 1, 4, 5, and 7 share substantial sequence similarity (≥50%) with established biosynthetic pathways for secondary metabolites. This similarity suggests that Bos-x6-28 may have the genetic capacity to produce metabolites resembling sporulation killing factor, the siderophore fengycin, carotenoids, and lichenysin-like compounds.

Conversely, the remaining biosynthetic gene clusters are either unannotated in existing biosynthetic databases or display very low similarity to known natural product pathways, implying that Bos-x6-28 might possess the genetic machinery to produce previously uncharacterized natural products. These findings underscore its potential as a promising source of novel bioactive compounds.

### 2.7. Antibacterial and Antitumor Activities of Strain Bos-x6-28

**Figure 6:**
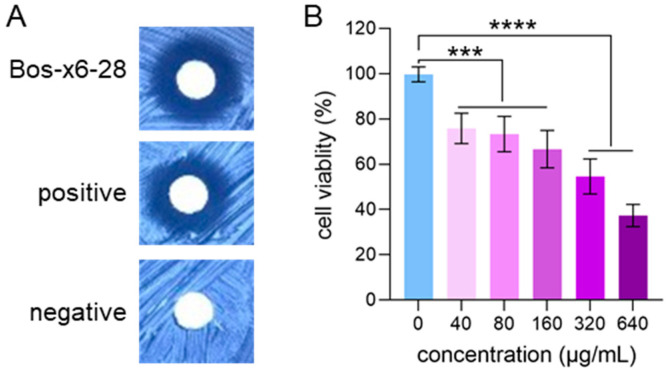
Bioactivity of secondary metabolites from strain Bos-x6-28. (**A**) Inhibitory activity against *B. subtilis*, with ampicillin as a positive control and methanol as a negative control. (**B**) Inhibitory activity against human liver cancer cells (HepG2), using DMSO as the blank control. Data are presented as mean ± SD, derived from three independent experiments conducted in triplicate. Statistical significance is indicated as *** *p* < 0.001 compared to control cells, and **** *p* < 0.0001 compared to control cells (Wilcoxon *t*-test).

The antibacterial efficacy of fermentation products derived from strain Bos-x6-28 was assessed against several pathogenic indicators, including *Staphylococcus aureus* ATCC 51650, *Bacillus subtilis* ATCC 23857, *Escherichia coli* ATCC 25922, and *Candida albicans* ATCC 10231. Results demonstrated that Bos-x6-28 exhibited potent inhibitory activity, specifically against *B. subtilis* (Figure 6A), with a minimum inhibitory concentration (MIC) of 50 μg·mL^−1^, while no inhibitory effects were observed against *S. aureus*, *E. coli*, or *C. albicans*.

In addition, the cytotoxic potential of Bos-x6-28 fermentation products against human hepatocellular carcinoma (HepG2) cells was evaluated using the CCK-8 assay. The findings indicated a dose-dependent inhibition of HepG2 cell proliferation over a concentration range of 40–640 μg/mL, with an IC_50_ value of 365.3 μg/mL (Figure 6B). These results suggest that Bos-x6-28 exhibits significant antitumor activity against HepG2 cells, highlighting its potential as a promising source of antineoplastic agents.

## 3. Discussion

The identification of novel bacterial species within the yak gut microbiota holds substantial implications, not only for deepening our understanding of microbial diversity and ecological function within the yak gastrointestinal ecosystem but also for the potential discovery of novel microbial-derived therapeutics. In this study, we applied polyphasic taxonomy [35,36,37]—a widely respected approach for new microbial species characterization—to establish the taxonomic status of a novel *Bacillus* species isolated from the feces of free-grazing yaks inhabiting high-altitude regions.

Comparative 16S rRNA gene sequence analysis indicated that strain Bos-x6-28 shares over 98.65% similarity with known species *B. xiamenensis* HYC-10^T^ and *B. zhangzhouensis* DW5-4^T^, surpassing the typical threshold for new species delineation [38]. Although the similarity exceeded the threshold typically used for identifying a novel species, and phylogenetic analysis indicated a close relationship with known strains, strain Bos-x6-28 formed an independent clade on the phylogenetic tree (Figure 1). This distinct branching pattern suggests that Bos-x6-28 may represent a novel species [39]. Additionally, the physiological and biochemical properties of Bos-x6-28 exhibited partial alignment with these related strains. Specifically, the cell membrane phospholipid profile of Bos-x6-28, comprising PE, PC, and DPG (Figure 3), is characteristic of the *Bacillus* genus [31]. However, Bos-x6-28 utilized a narrower spectrum of carbon sources than strain types HYC-10^T^ and DW5-4^T^ (Table 1), and its enzyme activity profile displayed moderate divergence. Particularly notable is the distinct cellular fatty acid composition of Bos-x6-28, which diverges substantially from its close relatives (Table 2), suggesting that this strain represents a potentially novel species within the *Bacillus* genus.

To further substantiate this classification, we conducted genomic analyses using dDDH and ANI, both of which are critical for bacterial species delineation when 16S rRNA similarity exceeds 98.65% [38]. For strain Bos-x6-28, dDDH and ANI comparisons with the closest related strains, HYC-10^T^ and DW5-4^T^, yielded values markedly below the thresholds for species identification, with maximum similarities of 54.7% and 86.71%, respectively (Table 4). Comparative genomic analyses against seven phylogenetically related strains provided additional evidence supporting Bos-x6-28 as a novel *Bacillus* species [40]. Based on these comprehensive polyphasic taxonomy findings, we propose the designation *Bacillus maqinnsis* sp. nov. Bos-x6-28 (ma.qin.nsis. N.L. fem. adj. *maqinnsis* of Ma-qin, a county in Qinghai, P. R. China, where the strain type was isolated) for this new species, following established nomenclatural conventions [41]. The strain has been deposited in the Marine Culture Collection of China (MCCC)] under the accession number MCCC 1K09379.

Using the antiSMASH online tool, we predicted the secondary metabolite biosynthetic potential of strain Bos-x6-28 (Table 5). The analysis revealed that this strain harbors the genetic capacity to produce a variety of compounds, including β-lactone derivatives, type III polyketides, siderophores, sactipeptides, and non-ribosomal peptide compounds. Notably, it exhibits the potential to synthesize bioactive siderophores such as fengycin and lichenysin. To date, over 30 β-lactone natural products have been identified, many of which demonstrate significant bioactivity against bacteria and fungi [42]. Fengycin, primarily produced by Gram-positive Bacillus spp., is an antimicrobial lipopeptide known for its potent antifungal activity, particularly against filamentous fungi, though it shows no effect on bacteria or yeast [43,44,45]. However, in our study, Bos-x6-28 fermentation extracts exhibited inhibitory effects against *B. subtilis*, suggesting that this strain may produce structurally unique derivatives of fengycin.

Research by Piewngam et al. demonstrated that fengycin can inhibit the colonization of *S. aureus* in mouse models [46]. Fengycin also exhibits notable antitumor properties, with studies showing selective activity against tumor cells and tissues. Extensive research has further indicated its potential in inhibiting the proliferation of human colon cancer HCT-15 cells [47] and in inducing cell cycle arrest at the G0/G1 phase, as well as promoting apoptosis in human lung cancer 95D cells [48], underscoring its substantial antitumor potential.

Lichenysin is another secondary metabolite synthesized by non-ribosomal peptide synthetase (NRPS) pathways [49]. This amphipathic compound, composed of a cyclic peptide of seven amino acids condensed with a β-hydroxy fatty acid side chain, exhibits stronger surfactant activity than surfactin [50,51]. Lichenysin has demonstrated a range of bioactivities, including antitumor [52], antiviral [53], neuroprotective, and anticancer effects [54], and is reported to contribute to flavor formation in traditional Chinese distilled spirits [55]. Additionally, the sporulation-killing factor (SKF) is a sactipeptide, a subclass of RiPPs. In *Bacillus* species, this molecule plays a critical role in the sporulation process and under nutrient-limiting conditions [56]. Its biosynthesis involves additional mechanisms that confer immunity against its toxicity. Previous studies have shown that Bacillus subtilis produces a spore-killing factor to eliminate non-sporulating sibling cells that are susceptible to these toxins. The synthesis of this compound helps maintain a small population of spores while preserving the majority of vegetative cells [57]. Additionally, the produced toxin can kill other microorganisms and provide nutrients to delay sporulation [58]. This mechanism may be associated with the adaptation of *Bacillus* strain Bos-x6-28 to the gut environment, where non-sporulating microorganisms coexist. A similar phenomenon has also been observed in *B. altitudinis* strain 19_A [59].

Our findings also show that strain Bos-x6-28 exhibits cytotoxicity against human hepatocellular carcinoma HepG2 cells, although the specific antitumor compounds responsible for this effect require further investigation. In addition, the genome of strain Bos-x6-28 contains genes that potentially encode secondary metabolites, including T3PKS and RRE-containing molecules, although these genes remain unannotated. This indicates that strain Bos-x6-28 has the potential to synthesize novel natural products with these structural features. Future studies will employ OSMAC (One Strain, Many Compounds) approaches and metabolomic techniques to identify these potential new molecules. Collectively, these results highlight the potential of strain Bos-x6-28 to synthesize bioactive natural products, positioning it as a valuable source for novel antibiotics and anticancer agents.

## 4. Materials and Methods

### 4.1. Strains

In this study, strain Bos-x6-28 was isolated from the feces of free-grazing yaks in Maqin County, Qinghai Province. The detailed fecal collection procedure has been previously reported by our group [23]. The isolation process was as follows: fecal samples were collected from 3-year-old, pasture-raised yaks that had not been treated with antibiotics in the previous 6 months. To minimize environmental contamination, the surface layer of freshly excreted feces was first removed, and approximately 1 g of the internal fresh feces was collected into sterilized 50 mL centrifuge tubes. Fecal samples were obtained from 10 different yaks and transported to the laboratory in a 4 °C vehicle-mounted refrigerator. The 10 samples were then pooled, and 1 g of the mixed fecal sample was added to 10 mL of sterile PBS buffer. The mixture was vortexed thoroughly and centrifuged at 1000 r/min for 1 min, and 100 μL of the supernatant were serially diluted (10^−1^, 10^−3^, 10^−5^, 10^−7^). From each dilution, 100 μL were evenly spread onto solid LB agar plates and incubated at 37 °C for 24–48 h. Individual colonies with distinct colors, sizes, and morphologies were selected from the plates and subjected to streak purification. The purified strains were then scaled up in liquid culture, harvested, and suspended in 30% glycerol before being stored at −80 °C for long-term preservation. The strain types *B. xiamenensis* HYC-10^T^ and *B. zhangzhouensis* DW5-4^T^ were obtained from the Marine Culture Collection of China (MCCC). All strains were stored at −80 °C in 20% glycerol in the Biopharmaceutical Laboratory of the College of Eco-Environmental Engineering, Qinghai University.

### 4.2. Morphological Observation

Strain Bos-x6-28 was inoculated on ISP2 medium (4 g yeast extract, 4 g glucose, 10 g malt extract, 6 g agar, 1 L ddH_2_O, pH 7.0) and incubated at 37 °C for 3 days for morphological observation using Gram staining under a light microscope (Eclipse 50i, Nikon, Tokyo, Japan), followed by ultrastructural analysis with field emission scanning electron microscopy (JSM-7900F, JEOL, Tokyo, Japan).

### 4.3. Physiological and Biochemical Testing

To determine the tolerance characteristics of strain Bos-x6-28 to temperature, pH, and salinity, it was inoculated into TSB medium (30 g tryptone soya broth, 1 L ddH_2_O, pH 7.0) and cultured at varying temperatures (10–40 °C, in 5 °C increments), pH values (4.0–10.0, at intervals of 1 pH unit), and NaCl concentrations (0–5%, in 1.0% increments, *w*/*v*) under 180 rpm shaking for 3 days. Growth was monitored by measuring OD_600_. Carbon and nitrogen source utilization was assessed by inoculating strain Bos-x6-28 on solid media containing different carbon [2.64 g (NH_4_)_2_SO_4_, 5.65 g K_2_HPO_4_, 2.38 g KH_2_PO_4_, 1 g MgSO_4_·7H_2_O, 0.0064 g CuSO_4_·5H_2_O, 0.0011 g FeSO_4_·7H_2_O, 0.0079 g MnCl_2_·4H_2_O, 0.0015 g ZnSO_4_·7H_2_O, 10 g carbon source, 6 g agar, 1 L ddH_2_O, pH 7.0] and nitrogen sources (1 g glucose, 1 g K_2_HPO_4_, 0.5 g MgSO_4_·7H_2_O, 0.5 g NaCl, 0.01 g FeSO_4_·7H_2_O, 20 g agar, 1 g nitrogen source, 1 L ddH_2_O, pH 7.0) and incubating at 28 °C and 180 rpm for 5 days. Carbon sources included D-mannitol, D-arabinose, rhamnose, D-galactose, inositol, fructose, sorbitol, dulcitol, mannose, glucose, xylose, and ribose. Nitrogen sources included threonine, alanine, proline, asparagine, serine, arginine, tyrosine, glutamate, and glycine. Additional characteristics, such as H_2_S production, starch hydrolysis, and lipase activity, were tested according to methods described in Bergey’s manual of determinative bacteriology [60]. Other enzymatic activities were assessed using API ZYM strips (bioMérieux, Craponne, France) following the manufacturer’s instructions.

### 4.4. Chemotaxonomic Characterization

Strain Bos-x6-28 was cultured in TSB at 28 °C and 180 rpm for 3 days, harvested by centrifugation at 6000 rpm for 10 min at 4 °C, and freeze-dried for chemical composition analysis. Following Hasegawa et al. [19], 50 mg of freeze-dried cells were hydrolyzed with 100 μL of 0.5 M HCl and 100 μL of 6 M HCl at 121 °C. The hydrolyzed samples were analyzed on a microcrystalline cellulose plate using solvent systems of ethyl acetate:pyridine:acetic acid:water (8:5:1:1.5, *v*/*v*), methanol:pyridine:acetic acid:water (5:0.5:0.125:2.5, *v*/*v*) for chromatographic separation to determine whole-cell hydrolysate sugars and amino acid composition.

Following the method described previously [61], the main phospholipid components were analyzed by grinding 100 mg of freeze-dried cells, adding 15 mL methanol, and heating in a boiling water bath for 10 min. After cooling, 10 mL of chloroform and 2% NaCl solution were added. The organic phase was concentrated at 37 °C and dissolved in a chloroform:methanol mixture (2:1, *v*/*v*). Phospholipids were then separated on GF_254_ silica gel plates using two solvent systems: chloroform:methanol:water (65:25:4, *v*/*v*) and chloroform:acetic acid:methanol:water (80:15:12:4, *v*/*v*), visualized with ninhydrin, anisaldehyde, and molybdophosphate. Menaquinone extraction followed the method of Xie et al. [62], in which 100 mg of freeze-dried cells were ground and extracted with 40 mL of a methanol:chloroform mixture (1:2, *v*/*v*) in the dark under 180 rpm shaking overnight. The supernatant was concentrated under reduced pressure at 40 °C and dissolved in 0.5 mL acetone. The extract was subjected to thin-layer chromatography on GF254 silica gel plates using hexane:ethyl ether (85:15, *v*/*v*) as the eluting solvent. Bands with an Rf of 0.8 were collected under UV light, dissolved in 0.5 mL acetone, filtered through a 0.22 μm filter, and analyzed by LC-MS/MS, a UHPLC 1290 system (Agilent Technologies, Santa Clara, CA, USA) coupled to a Q Exactive Orbitrap MS (Thermo, Waltham, MA, USA). Chromatographic conditions: The analysis was performed on an ACQUITY UPLC^®^ HSS T3 column (2.1 mm × 100 mm, 1.8 µm, Waters, Milford, MA, USA). The mobile phase consisted of 0.1% formic acid in water (A) and methanol (B), with a gradient elution program as follows: 0 min, 1% B; 3 min, 1% B; 10 min, 20% B; 15 min, 40% B; 25 min, 55% B; 35 min, 70% B; 40 min, 85% B; and 45 min, 100% B. The injection volume was 1 μL, with a flow rate of 0.4 mL/min and a column temperature maintained at 30 °C. Mass spectrometry conditions: Mass spectrometric detection was conducted using a quadrupole/orbitrap mass spectrometer equipped with an electrospray ionization (ESI) source operating in positive ion mode. The resolution was set to 70,000 with a scan range of 100–1500 *m*/*z*. For dd-MS2 (data-dependent MS/MS) acquisition, the resolution was 17,500. The spray voltage was 3.5 kV, with a sheath gas flow rate of 13.5 L·min^−1^, an auxiliary gas flow rate of 4.5 L·min^−1^, a capillary temperature of 320 °C, and an auxiliary gas heater temperature of 375 °C.

Additionally, the fatty acid composition of freeze-dried cells was determined using the MIDI Sherlock Microbial Identification System (version 6.0B). Fatty acid methyl esters were prepared by saponification, methylation, and extraction following the standard MIDI protocol and analyzed using a gas chromatograph (6850 GC, Agilent Technologies, Santa Clara, CA, USA) with the Microbial Identification System TSBA6.0 database for identification [63]. Gas chromatography conditions: The analysis was performed using an HP-ULTRA2 column (30.0 m × 0.25 mm × 0.25 μm. Agilent Technologies, Santa Clara, CA, USA). The injection volume was 1 μL, with an inlet temperature of 250 °C. The temperature program was as follows: the initial temperature was set at 190 °C, then increased at a rate of 10 °C·min^−1^ to 285 °C, followed by a further increase at 60 °C·min^−1^ to 310 °C. The temperature of flame ionization detector was maintained at 260 °C. The flow rates for the gases were as follows: hydrogen at 30 mL/min, air at 400 mL·min^−1^, and nitrogen (makeup gas) at 30 mL·min^−1^.

### 4.5. 16S rRNA Gene Sequence Analysis

To further clarify the taxonomic position of strain Bos-x6-28, we initially amplified, sequenced, and analyzed the phylogenetic relationships of the 16S rRNA gene. Genomic DNA of Bos-x6-28 was extracted using the CTAB method, and amplification was performed using universal bacterial primers 27F (5′-AGAGTTTGATCCTGGCTCAG-3′) and 1492R (5′-TACGGCTACCTTGTTACGACTT-3′). The PCR reaction system included: 0.5 μL each of primers 27F and 1492R (10 μmol·L^−1^), 12.5 μL of Premix Taq (enzyme activity 0.05 U/μL), 2 μL of template DNA (concentration 56 ng/μL), and 9.5 μL of ddH_2_O, totaling 25 μL. The amplification conditions were as follows: initial denaturation at 95 °C for 3 min; 34 cycles of 95 °C for 45 s, 56 °C for 45 s, and 72 °C for 90 s; and a final extension at 72 °C for 10 min. PCR products were purified and sequenced by GENEWIZ Biotechnology Co Ltd (Suzhou, China) to provide insights into the phylogenetic relationship of Bos-x6-28 with closely related species. The resulting sequences were aligned and compared in the EzBioCloud database (https://www.ezbiocloud.net/, accessed on 5 June 2024) to retrieve closely related strain-type sequences, and a phylogenetic tree was constructed using the neighbor-joining method with 1000 bootstrap replicates to ensure robustness of the analysis.

### 4.6. Whole-Genome Sequencing Analysis

For whole-genome sequencing, Bos-x6-28 was sequenced on the PacBio platform by Biomarker Technologies (Beijing, China), with subsequent filtering of low-quality reads. The qualified reads were assembled using Canu v1.5 software [64], followed by gene prediction with Prodigal V2.6.3 [65]. Visualizations of the assembled genome were created using Circos v0.66 [66], and the genome sequence was deposited in GenBank under accession numbers CP159358–CP159360. To explore the biosynthetic potential of secondary metabolites, antiSMASH analysis was performed, identifying potential gene clusters involved in the synthesis of bioactive compounds.

Further classification was supported by dDDH and ANI calculations. These metrics were obtained through the GGDC (https://ggdc.dsmz.de/, accessed on 5 June 2024) and JSpecies (https://jspecies.ribohost.com/jspeciesws/, accessed on 5 June 2024) platforms, respectively. To confirm the taxonomic placement of Bos-x6-28 and its distinctiveness from related strains, comparative genomic analysis was also performed using anvi’o v8.0 [40], comparing Bos-x6-28 (CP159358) with seven closely related strain types [*B. safensis* subsp. *safensis* FO-36b^T^ (CP010405.1), *B. safensis* subsp. *osmophilus* BC09^T^ (QBHN01000049.1), *B. australimaris* NH7I-1^T^ (LGYN01000001.1), *B. pumilus* NCTC10337^T^ (LT906438.1), *B. zhangzhouensis* DW5-4^T^ (JOTP01000001.1), *B. altitudinis* 41KF2b^T^ (ASJC01000001.1), and *B. xiamenensis* HYC-10^T^ (AMSH01000001.1)].

### 4.7. Antibacterial and Cytotoxicity Testing of Fermentation Products from Bos-x6-28

Strain Bos-x6-28 was inoculated into 5 mL of LB liquid medium and cultured at 37 °C with shaking at 200 rpm for 3 days to prepare the seed culture. This seed culture was then transferred at 5% volume into 100 mL of LB medium, incubated for 4 days, followed by the addition of 10% sterilized macroporous adsorption resin (Diaion HP20, Mitsubishi Chemical Group Corporation, Tokyo, Japan), and continued culturing for an additional 3 days. The fermentation product was eluted from the resin with five volumes of methanol and concentrated using a rotary evaporator to obtain the extract. The experiment was repeated three times, and three parallel fermentation extracts were prepared for subsequent bioactivity assays. Antimicrobial activity of the extracts was assessed against *S. aureus* ATCC 51650, *B. subtilis* ATCC 23857, *E. coli* ATCC 25922, and *C. albicans* ATCC 10231 using the disc diffusion method, with amphotericin B, sodium ampicillin, and azithromycin as positive controls, while methanol was used as a negative control. The concentration of the fermentation products (dissolved in methanol) was set at 100 mg·mL^−1^, and the concentration of the positive control drugs was set at 50 μg·mL^−1^. The detailed procedure was as follows: sterile filter paper discs (6 mm in diameter) were placed on the surface of agar plates inoculated with the test microorganisms. A precise volume of 2 μL of the sample solution was applied to the center of each disc, allowing it to diffuse naturally. After the solvent had evaporated, the plates were incubated at 37 °C for 48 h. The presence or absence of an inhibition zone was observed, and the diameter of the inhibition zones was measured to preliminarily assess the antimicrobial activity of the fermentation products. MIC was determined by a micro-dilution method we previously reported [67]. Briefly, the extract was dissolved in methanol to a stock concentration of 4 mg·mL^−1^. After 12 h of culture at 37 °C and 180 rpm, the four indicator strains were diluted 1000-fold with sterile LB medium. Diluted bacterial cultures (100 μL) were added to each well of a 96-well plate, followed by varying concentrations of the extract to achieve final concentrations ranging from 0.19 to 100 μg·mL^−1^ in triplicate. Positive controls included amphotericin B, sodium ampicillin, and azithromycin, with methanol as a negative control. Plates were incubated at 37 °C for 16 h, and MIC values were defined as the lowest concentration resulting in the clearest medium.

For cytotoxicity testing, HepG2 cells in logarithmic growth phase were digested with 0.25% trypsin and resuspended in MEM medium at a density of 1 × 10^5^ cells·mL^−1^. A 100 μL cell suspension was added to each well of a 96-well plate and incubated at 37 °C in 5% CO_2_ for 24 h. Test compounds were dissolved in DMSO to prepare stock solutions, which were further diluted in MEM to obtain working solutions at concentrations of 40, 80, 160, 320, and 640 μg·mL^−1^. After cell attachment, the original medium in the 96-well plate was replaced with MEM containing different concentrations of test compounds (100 μL per well), with each concentration in six replicates. Cell viability was evaluated by the CCK-8 assay, and absorbance was measured at 450 nm using a microplate reader. Cell survival rate was calculated as follows: cell viability (%) = (absorbance of test group/absorbance of control group) × 100%. The IC_50_ values were determined using GraphPad Prism 8.0 software.

## 5. Conclusions

In this study, we conducted a comprehensive polyphasic taxonomic analysis of a novel Bacillus species isolated from the feces of free-grazing yaks in high-altitude regions, examining its morphological, physiological, biochemical, chemical, and molecular characteristics. The results indicate that this strain shares the closest phylogenetic relationship with *B. xiamenensis* HYC-10^T^ and *B. zhangzhouensis* DW5-4^T^. However, dDDH and ANI analyses, along with comparative genomic assessments, confirm that this strain represents a distinct species within the *Bacillus* genus, for which we propose the name *Bacillus maqinnsis* Bos-x6-28. Furthermore, genome analysis suggests that strain Bos-x6-28 possesses the biosynthetic potential to produce bioactive compounds, including derivatives of fengycin and lichenysin. Preliminary in vitro activity assays reveal that Bos-x6-28 exhibits antimicrobial and antitumor activity, highlighting its potential as a source for novel microbial therapeutics. However, further research is required to identify and characterize the specific active compounds responsible for these bioactivities.

## Figures and Tables

**Table 1:** Comparison of physiological and biochemical characteristics between the strain Bos-x6-28 and mostly similar strain types.

Items	Bos-x6-28	*B. xiamenensis* HYC-10^T^	*B. zhangzhouensis* DW5-4^T^
Growth Temperature Range (°C)	10–40	10–40	10–40
NaCl Tolerance Range (%)	0–5	0–5	0–5
pH Range	4–10.0	4–10.0	4–10
D-mannitol	−	−	+
D-arabinose	−	+	+
Rhamnose	−	+	−
D-galactose	+	+	+
Inositol	+	−	−
Fructose	+	+	+
Sorbitol	−	+	−
Uranose	+	+	+
D-mannose	+	+	+
Glucose	+	+	+
Xylose	+	+	+
Ribose	+	+	+
Threonine	+	+	+
Alanine	+	+	+
Proline	+	+	+
Asparagine	+	+	+
Serine	+	+	+
Arginine	+	+	+
Tyrosine	+	+	+
Glutamate	+	+	+
Glycine	+	+	+
H_2_S production	−	−	−
Starch hydrolysis	−	−	−
Twain-20	+	+	+
Twain-60	+	+	+
Twain-80	+	+	+
Alkaline phosphatase	+	+	+
Esterase (C4)	+	+	+
Esterase lipase (C8)	+	+	+
Esterase (C14)	+	+	+
Leucine arylamidase	+	+	+
Valine arylamidase	+	+	+
Cystine arylaminase	+	+	+
Trypsin	+	+	+
Chymotrypsinalpha	+	+	+
Acid phosphatase	+	−	+
Naphthol-AS-BI-Phosphohydrolase	−	−	+
α-galactosidase	+	−	+
β-galactosidase	−	−	+
β-glucuronidase	−	−	−
α-glucosidase	+	−	+
β-glucosidase	−	−	+
N-acetyl-glucosidase	−	−	−
α-mannosidase	+	−	+
α-fucosidase	+	+	+

+, positive; −, negative.

**Table 2:** Comparison of polar lipids in the strain BoS-x6-28 and mostly similar strain types.

Fatty Acid	Bos-x6-28	*B. xiamenensis* HYC-10^T^	*B. zhangzhouensis* DW5-4^T^
C_14:0_	8.75	tr	tr
C_16:0_	28.0	6.1	1.6
C_16:1_*ω*7*c* alcohol	nd	tr	tr
C_18:0_	tr	1.2	nd
C_18:1_*ω*9*c*	nd	tr	nd
Iso-C_13:0_	3.15	tr	tr
Iso-C_14:0_	tr	1.8	1.6
Iso-C_15:0_	5.49	39.1	45.0
Iso-C_16:0_	tr	5.1	3.0
Iso-C_17:0_	2.55	13.1	4.5
Iso-C_17:1_*ω*10*c*	tr	tr	tr
Anteiso-C_15:0_	4.61	22.7	35.6
Anteiso-C_17:0_	1.36	5.8	4.6

nd, not detected; tr, trace amount (<1.0%).

**Table 3:** Summary of Bos-x6-28 strain sequencing data.

Iterms	Value
Genome size (bp)	3,329,571
Number of Scaffolds	3
GC content (%)	40.22
Coding genes	3353
Total of gene length (bp)	2,841,843
Average gene length (bp)	847
Total length of repeated sequences (bp)	4869
Number of 5s/16s/23s rRNA	8/8/8
Number of tRNA	88
Number of predicted CRISPR sequence	6
Number of gene islands	4

**Table 4:** Comparison of the molecular characteristics between the strain Bos-x6-28 and the most similar strain types.

Strain Types	16S rRNA Gene Similarity (%)	dDDH (%)	ANI (%)
*B. xiamenensis* HYC-10^T^	98.91	52.1	85.88
*B. zhangzhouensis* DW5-4^T^	98.91	54.7	86.71

**Table 5:** Prediction of biosynthetic gene clusters encoding secondary metabolites in the strain Bos-x6-28 genome.

NO.	Types of Gene Clusters	Location of Gene Clusters	Most Similar Known Cluster	Similarity (%)
1	β-lactone	1/24,340	/	/
2	Ladderane	117,458/159,914	S-layer glycan	20%
3	T3PKS	455,994/497,091	/	/
4	NRPS	593,742/622,162	Fengycin	53%
5	Siderophore	1,234,652/1,260,666	Carotenoid	50%
6	RRE-containing	1,404,864/1,424,767	/	/
7	NRPS	1,798,336/1,882,055	Lichenysin	78%
8	Sactipeptide	1,970,815/1,993,765	Sporulation Killing factor	100%

/, unannotated.

## Data Availability

The original contributions presented in this study are included in the article/supplementary material. Further inquiries can be directed to the corresponding authors.

## Supplementary Materials

The following supporting information can be downloaded at: https://www.mdpi.com/article/10.3390/antibiotics13121238/s1, Figure S1: The growth characteristics of strain Bos-x6-28 under varying temperature (A), pH (B), and salinity (C) conditions; Figure S2: Mass spectrometry profile of menaquinone in strain Bos-x6-28; Table S1: Comparative genomic information of strain Bos-x6-28 and other closely related *Bacillus* species.
