# Supplementary material for: Identification and Bioactivity Analysis of a Novel Bacillus Species, B. maqinnsis sp. nov. Bos-x6-28, Isolated from Feces of the Yak (Bos grunniens)
Source: Antibiotics (Basel). 2024 Dec 23;13(12):1238. doi: 10.3390/antibiotics13121238 (PMC11672612; doi:10.3390/antibiotics13121238)
Supplement: Supplementary file 1 [file antibiotics-13-01238-s001.zip › Supplementary Figures.pdf]

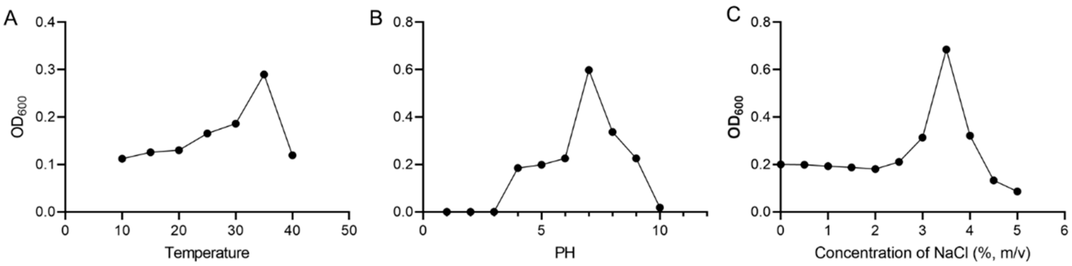

Figure S1. The growth characteristics of strain Bos-x6-28 under varying temperature (A), pH (B), and salinity (C) conditions.

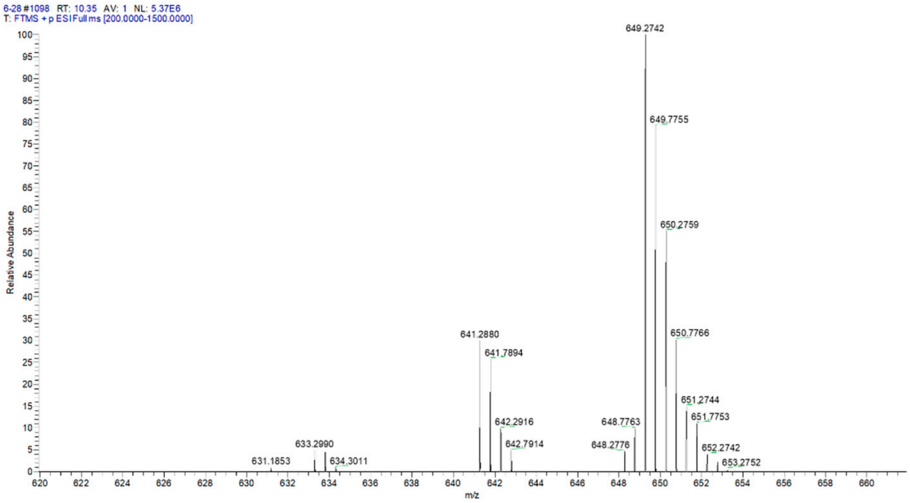

Figure S2. Mass spectrometry profile of menaquinone in strain Bos-x6-28.
